# Deep-Red and Near-Infrared Iridium Complexes with Fine-Tuned Emission Colors by Adjusting Trifluoromethyl Substitution on Cyclometalated Ligands Combined with Matched Ancillary Ligands for Highly Efficient Phosphorescent Organic Light-Emitting Diodes

**DOI:** 10.3390/molecules27010286

**Published:** 2022-01-04

**Authors:** Shuonan Chen, Hai Bi, Wenjing Tian, Yu Liu

**Affiliations:** 1 State Key Laboratory of Supramolecular Structure and Materials, College of Chemistry, Jilin University, Changchun 130012, China; chensn19@mails.jlu.edu.cn; 2 Jihua Laboratory, 13 Nanpingxi Road, Foshan 528200, China

**Keywords:** deep-red and near-infrared, iridium complexes, phosphorescent, organic light-emitting diodes (OLEDs)

## Abstract

Six novel Ir(C^N)_2_(L^X)-type heteroleptic iridium complexes with deep-red and near-infrared region (NIR)-emitting coverage were constructed through the cross matching of various cyclometalating (C^N) and ancillary (LX) ligands. Here, three novel C^N ligands were designed by introducing the electron-withdrawing group CF_3_ on the ortho (o-), meta (m-), and para (p-) positions of the phenyl ring in the 1-phenylisoquinoline (piq) group, which were combined with two electron-rich LX ligands (dipba and dipg), respectively, leading to subsequent iridium complexes with gradually changing emission colors from deep red (≈660 nm) to NIR (≈700 nm). Moreover, a series of phosphorescent organic light-emitting diodes (PhOLEDs) were fabricated by employing these phosphors as dopant emitters with two doping concentrations, 5% and 10%, respectively. They exhibited efficient electroluminescence (EL) with significantly high EQE values: >15.0% for deep red light0 (*λ*_max_ = 664 nm) and >4.0% for NIR cases (*λ*_max_ = 704 nm) at a high luminance level of 100 cd m^−2^. This work not only provides a promising approach for finely tuning the emission color of red phosphors via the easily accessible molecular design strategy, but also enables the establishment of an effective method for enriching phosphorescent-emitting molecules for practical applications, especially in the deep-red and near-infrared region (NIR).

## 1. Introduction

Organic near-infrared (NIR) materials and their light-emitting devices were developed rapidly since they are widely used in information-secured displays and night-vision/photodynamic therapy/signal processing devices [1,2,3]. Among them, thermally activated delayed fluorescence (TADF) organics [2] and phosphorescent transition-metal complexes based on the platinum (II) and osmium (II) family [3] seem to be ideal candidates for efficient NIR organic light-emitting diodes (OLEDs) due to both 25% singlet and 75% triplet excited states being converted into light emissions, which can generally achieve external quantum efficiencies (EQEs) of over 10%. Nevertheless, some negative factors related to these above-mentioned materials (or devices), such as expensive raw materials, several additional auxiliary layers required, adopting mixed host systems based on precise-control concentrations, etc., restrict their further application in NIR-electroluminescent (EL) devices [4,5]. Therefore, it is necessary to explore the challenge of long wavelength-emitting systems with higher and/or more balanced comprehensive characteristics. Currently, as the most prominent class of molecular phosphors, the luminescent cyclometalated iridium (III) complex system has demonstrated almost perfect EL performance in terms of its luminance, efficiency, and color emission from the blue (*λ*_max_ ≈ 470 nm) to saturated red (*λ*_max_ ≈ 650 nm) regions, thereby having a significant impact on display technologies. Normally, lower-energy regions (e.g., *λ*_max_ > 650 nm) of the spectrum usually have lower-middle quantum yields, according to the energy gap law. However, for heteroleptic iridium complexes with the C^N = cyclometalating ligand and L^X = ancillary ligand type [6,7,8,9,10,11], it has been demonstrated that introducing groups or molecules with obvious electron-rich character into the Ir (III) complexes as LX ligands can greatly affect the electronic structures of the complex. Thus, by adjusting their opto-electronic properties involving luminous color, phosphorescence quantum yields, and even their charge-transporting characteristics, it is possible to enhance their EL device performance relative to corresponding analogs based on acetylacetonate (acac) as an ancillary ligand or homoleptic tris-cyclometalated fac-Ir(C^N)_3_ complexes. In general, the preparing processes of Ir(C^N)_2_(L^X)-typed complexes are usually cheaper and easier than those of the homoleptic cases, and in the former cases, C^N and L^X provide two relatively independent channels to adjust the opto-electronic characteristics of Ir (III) complexes, their emission color, frontier orbitals, and excited states can be precisely and finely tuned by coordinately altering and matching different C^N/L^X combinations. This strategy avoids the complex optimization route through contraction or extension of the π-conjugation system, and their steric and/or electronic constraints may increase the difficulty of preparing chloride-bridged [Ir(C^N)_2_(*μ*-Cl)]_2_ complexes, which are necessary precursors in the common synthesis of iridium-based phosphorescent molecules [12]. Our group developed an effective molecular design strategy by using a series of amidinate/guanidinate groups as ancillary ligands with significant electron-donating properties and constructed a four-membered Ir–N–C–N backbone-based type on the Ir (III) complex with known C^N ligands, which exhibited highly efficient electrophosphorescent emission covering almost the entire visible region with a wavelength range from 470 nm to 660 nm [13,14,15,16,17]. More recently, we reported the effect of structural modifications by anchoring two electron-withdrawing groups fluoride (F) and trifluoromethyl (CF_3_) on the phenyl ring of the classical C^N ligand 2-phenylpyridine (ppy), and these substituents effectively induced varying degrees of change in the HOMO (highest occupied molecular orbital) and the LUMO (lowest unoccupied molecular orbital) levels of the target complexes, leading to green-emitting (*λ*_max_ = 545 nm) and orange-emitting (*λ*_max_ = 581 nm) light, respectively [18].

In this work, three C^N ligands were constructed by introducing the electron-withdrawing group CF_3_ on the ortho (o-), meta (m-), and para (p-) positions of the phenyl ring in the 1-phenylisoquinoline (piq) group, which combines crossly with two electron-rich ancillary ligands (*N*,*N*′-diisopropylbenzamidinate and *N*,*N*′-diisopropyl- diisopropylguanidinate, abbreviated as dipba and dipg, respectively) [17,18] and constructed six heteroleptic Ir (III) complexes with gradually changing color emissions from deep red (≈660 nm) to NIR (≈700 nm). Our research provides insight into the influence of different pairs of C^N and LX ligands on the electrochemical, photophysical, and EL properties. Moreover, a series of well-matched C^N/LX combinations ensured that each complex presented high photoluminescence (PL) emission efficiency as well as sufficient charge-transporting ability for holes and electrons. As a result, these new phosphors were successfully employed as dopant emitters in a common host bis(10-hydroxybenzo(h)quinolinato)beryllium complex (Bebq_2_) with two doping concentrations of 5% and 10%, respectively, to achieve a series of PhOLEDs with scattered EL emission wavelengths between 664 and 704 nm, achieving significantly high EQE values, >15.0% for deep red (*λ*_max_ = 664 nm) devices and >4.0% for NIR (*λ*_max_ = 704 nm) cases, which were obtained at a fairly high luminance level of 100 cd m^−^^2^. However, although these are not extremely high efficiency levels, this strategy can be indeed easily carried out and is feasible for fine tuning the emission color of long-wavelength phosphors, and newer phosphorescent-emitting molecules possessing desirable optoelectronic characteristics in the deep-red and NIR region are, therefore, expected.

## 2. Results and Discussion

### 2.1. Synthesis

**Scheme 1:**
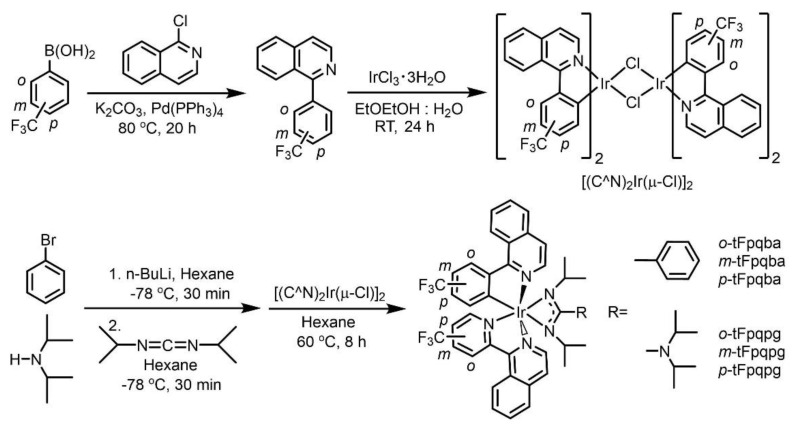
Synthesis and chemical structures of the iridium complexes.

The general synthetic procedure and chemical structure for the six novel complexes of type (C^N)_2_Ir(LX) (C^N = 1-(2-(trifluoromethyl)phenyl)isoquinoline (*o*-CF_3_piq), 1-(3-(trifluoromethyl)phenyl)isoquinoline (*m*-CF_3_piq) and 1-(4-(trifluoromethyl)phenyl) isoquinoline (*p*-CF_3_piq) and LX = *N*,*N*′-diisopropylbenzamidinate (dipba) and *N*,*N*′ -diisopropyl-diisopropylguanidinate (dipg), respectively) is described in Scheme 1. Anhydrous tetrahydrofuran and hexane were pre-distilled with sodium benzophenone ketyl under a nitrogen atmosphere. Other organic solvents and materials were obtained from commercial suppliers and were used without further refinement. Round-bottom flasks, magnetic stirring bars, and syringe needles were oven-dried. All synthetic processes were performed using standard Schlenk techniques in a nitrogen atmosphere. Three new C^N ligands, *o*-CF_3_piq, *m*-CF_3_piq, and *p*-CF_3_piq, were synthesized according to the classical Suzuki-coupling reaction. The synthesis of three *μ*-chloro-bridged dimers [(C^N)_2_Ir(*μ*-Cl)]_2_ and the final six complexes is similar to the procedure described in the literature [12,15,16]. The above reactions were monitored with thin-layer chromatography (TLC) at wavelengths of 254 nm and 365 nm under UV light. Mass spectra were measured on a GC-MS (Thermo Fisher Technology Co., Ltd., Shanghai, China) or MALDI-TOF-MS mass spectrometer (Bruker Technology Co., Ltd., Beijing, China). ^1^H and ^13^C NMR spectra were recorded on an AVANCE III 500 MHz spectrometer (Bruker) using CDCl_3_ as the solvent and tetramethylsilane (TMS) as the internal reference. Elemental analyses were carried out on a Vario micro cube (Elementar).

1-(2-(Trifluoromethyl)phenyl)isoquinoline (*o*-CF_3_piq). 1-Chloroisoquinoline (2.5 g, 15 mmol) and (2-(trifluoromethyl)phenyl)boronic acid (3.4 g, 18 mmol) were dissolved in a mixture of tetrahydrofuran (50 mL), ethanol (25 mL), and potassium carbonate aqueous solution (2 M, 40 mL). Then, tetrakis(triphenylphosphine)palladium (0) (90 mg, 0.08 mmol) was quickly added. The reaction mixture was stirred at 80 °C for 20 h under a nitrogen atmosphere. After cooling, the mixture was extracted with ethyl acetate (3 × 20 mL) and washed with brine. The solvent was removed, and the residue was purified by column chromatography on silica gel (eluent: dichloromethane/petroleum ether = 1:2 *v*/*v*) to afford ***o*-CF_3_piq** as yellowish oily liquid (3.3 g; yield: 82%). MS (EI): *m*/*z* 273.40 (M^+^). ^1^H NMR (500 MHz, CDCl_3_) *δ* [ppm] 8.63 (d, *J* = 5.7 Hz, 1H), 7.92 (d, *J* = 8.3 Hz, 1H), 7.88 (d, *J* = 7.6 Hz, 1H), 7.75 (d, *J* = 6.1 Hz, 1H), 7.72 (dd, *J* = 8.0, 1.4 Hz, 1H), 7.69 (d, *J* = 7.2 Hz, 1H), 7.65 (t, *J* = 7.5 Hz, 1H), 7.55 (d, *J* = 8.0 Hz, 1H), 7.53–7.49 (m, 1H), 7.48 (d, *J* = 7.4 Hz, 1H).

1-(3-(Trifluoromethyl)phenyl)isoquinoline (*m*-CF_3_piq) was synthesized similarly to *o*-CF_3_piq, affording a colorless transparent oily liquid (2.8 g; yield: 67%). MS (EI): *m*/*z* 272.55 (M^+^). ^1^H NMR (500 MHz, CDCl_3_) *δ* [ppm] 8.66 (d, *J* = 5.7 Hz, 1H), 8.04 (d, *J* = 8.5 Hz, 1H), 8.02 (s, 1H), 7.94 (t, *J* = 9.1 Hz, 2H), 7.82–7.77 (m, 1H), 7.75 (t, *J* = 6.9 Hz, 2H), 7.70 (t, *J* = 7.7 Hz, 1H), 7.62 (t, *J* = 7.7 Hz, 1H).

1-(4-(Trifluoromethyl)phenyl)isoquinoline (*p*-CF_3_piq) was synthesized similarly to ***o*-CF_3_piq**, affording a white solid (3.2 g; yield: 79f%). MS (EI): *m*/*z* 274.43 (M+H^+^). ^1^H NMR (500 MHz, CDCl_3_) *δ* [ppm] 8.66 (d, *J* = 5.7 Hz, 1H), 8.06 (d, *J* = 8.5 Hz, 1H), 7.95 (d, *J* = 8.2 Hz, 1H), 7.85 (q, *J* = 8.4 Hz, 4H), 7.78–7.75 (m, 1H), 7.74 (d, *J* = 5.6 Hz, 1H), 7.63–7.58 (m, 1H).

Synthesis of (*o*-CF_3_piq)_2_Ir(dipba) (abbreviated as *o*-tFpqba). A mixture of ligand *o*-CF_3_piq (2.0 g, 7.5 mmol) and IrCl_3_·3H_2_O (1.1 g, 3 mmol), 15 mL of 2-ethoxyethanol, and 5 mL of H_2_O was refluxed for 24 h. After the reaction finished, the mixture was poured into water and the formed precipitate was filter washed by water and cold methanol ether several times to yield a dark red-brown solid. The crude product was assumed to be the chloro-bridged dimer, without any further purification. In a 50 mL flask, *n*-BuLi (0.42 mL, 2.5 M in hexane) was added dropwise to 10 mL anhydrous tetrahydrofuran solution of bromobenzene (0.16 g, 1 mmol) at −78 °C. The reaction mixture was stirred for 30 min. Then, *N*,*N*′-diisopropylcarbodiimide (DIC, 126 mg, 1 mmol) was added dropwise. The reaction mixture was stirred for another 30 min. The mixture was added dropwise to an anhydrous tetrahydrofuran solution of chloro-bridged dimer (385 mg, 0.25 mmol). After stirring at 60 °C for 8 h, the reaction was quenched with water, extracted with ethyl acetate, dried over anhydrous Na_2_SO_4_, and removed the solvent. The crude product was purified by vacuum sublimation to obtain a brown-black powder (160 mg; yield: 34%). ^1^H NMR (500 MHz, CDCl_3_) *δ* [ppm]: 9.29 (d, *J* = 6.4 Hz, 2H), 8.32 (d, *J* = 8.3 Hz, 2H), 8.01 (d, *J* = 8.0 Hz, 2H), 7.75 (d, *J* = 6.5 Hz, 2H), 7.64 (dd, *J* = 13.8, 6.2 Hz, 4H), 7.50–7.46 (m, 2H), 7.42 (d, *J* = 7.2 Hz, 1H), 7.37 (d, *J* = 7.4 Hz, 2H), 7.17 (d, *J* = 7.3 Hz, 2H), 6.65–6.61 (m, 2H), 5.62 (d, *J* = 7.6 Hz, 2H), 3.39–3.34 (m, 2H), 0.48 (d, *J* = 6.3 Hz, 6H), 0.19 (d, *J* = 6.2 Hz, 6H). MS (MALDI-TOF, *m*/*z*): [M^+^] calcd, 940.26; found, 939.69. Anal. Calcd for C45H37F6IrN4 (%): C, 57.50; H, 3.97; N, 5.96. Found: C, 57.48; H, 3.99; N, 5.97.

Synthesis of (*o*-CF_3_piq)_2_Ir(dipg) (abbreviated as *o*-tFpqpg). The synthesis process and amount used were similar to *o*-tFpqba using diisopropylamine instead of bromobenzene to obtain a dark-brown powder (175 mg; yield: 36%). ^1^H NMR (500 MHz, CDCl_3_) *δ* [ppm] 9.14 (d, *J* = 6.3 Hz, 2H), 8.27 (d, *J* = 8.4 Hz, 2H), 7.95 (d, *J* = 8.1 Hz, 2H), 7.71 (t, *J* = 7.5 Hz, 2H), 7.64 (d, *J* = 6.3 Hz, 2H), 7.60 (t, *J* = 7.6 Hz, 2H), 7.16 (d, *J* = 7.5 Hz, 2H), 6.61 (t, *J* = 7.6 Hz, 2H), 5.52 (d, *J* = 7.5 Hz, 2H), 3.92 (dt, *J* = 12.6, 6.3 Hz, 2H), 3.58 (dt, *J* = 13.3, 6.6 Hz, 2H), 1.32 (d, *J* = 6.7 Hz, 6H), 1.29 (d, *J* = 6.6 Hz, 6H), 0.67 (d, *J* = 6.3 Hz, 6H), 0.25 (d, *J* = 6.3 Hz, 6H). ^13^C NMR (126 MHz, CDCl_3_) *δ* [ppm] 170.99, 169.29, 159.97, 143.19, 142.10, 134.73, 133.99, 130.49, 129.2~130.0 (-CF_3_, q, *J* = 0.24 Hz), 128.51, 128.25, 128.08, 126.78, 125.86, 120.42, 120.11, 49.09, 47.48, 24.44, 24.01, 23.81, 22.96. MS (MALDI-TOF, *m*/*z*): [M^+^] calcd, 963.33; found, 963.85. Anal. Calcd for C45H46F6IrN5 (%): C, 56.12; H, 4.81; N, 7.27. Found: C, 56.14; H, 4.80; N, 7.25.

Synthesis of (*m*-CF_3_piq)_2_Ir(dipba) (abbreviated as *m*-tFpqba). This complex was collected as a dark-brown powder, according to the same procedure for *o*-tFpqba (183 mg; yield: 39%). ^1^H NMR (500 MHz, CDCl_3_) δ 9.45 (d, *J* = 6.4 Hz, 2H), 8.95–8.90 (m, 2H), 8.41 (s, 2H), 8.09–8.04 (m, 2H), 7.86–7.79 (m, 4H), 7.74 (d, *J* = 6.4 Hz, 2H), 7.48 (t, *J* = 7.3 Hz, 2H), 7.42 (t, *J* = 7.3 Hz, 1H), 7.38–7.31 (m, 2H), 6.84 (d, *J* = 8.0 Hz, 2H), 6.47 (d, *J* = 8.1 Hz, 2H), 3.30 (dq, *J* = 12.3, 6.1 Hz, 2H), 0.67 (d, *J* = 6.3 Hz, 6H), −0.12 (d, *J* = 6.3 Hz, 6H). ^13^C NMR (126 MHz, CDCl_3_) *δ* [ppm] 175.32, 169.02, 158.92, 149.74, 143.03, 136.66, 136.38, 130.77, 129.1~129.9 (-CF_3_, q, *J* = 0.24 Hz), 129.12, 128.44, 128.36, 128.32, 128.08, 127.82, 127.39, 126.57, 126.43, 120.81, 116.32, 48.27, 24.69, 24.48. MS (MALDI-TOF, *m*/*z*): [M^+^] calcd, 940.26; found, 939.73. Anal. Calcd for C45H37F6IrN4 (%): C, 57.50; H, 3.97; N, 5.96. Found: C, 57.52; H, 3.95; N, 5.97.

Synthesis of **(*m*-CF_3_piq)_2_Ir(dipg)** (abbreviated as ***m*-tFpqpg**)**.** This complex was collected as a dark-brown powder, according to the same procedure for ***o*-tFpqpg** (211 mg, Yield: 44%). ^1^H NMR (500 MHz, CDCl_3_) *δ* [ppm] 9.26 (d, *J* = 6.4 Hz, 2H), 8.87 (dd, *J* = 6.1, 3.5 Hz, 2H), 8.38 (s, 2H), 8.04–7.99 (m, 2H), 7.82–7.76 (m, 4H), 7.61 (d, *J* = 6.4 Hz, 2H), 6.82 (d, *J* = 8.0 Hz, 2H), 6.40 (d, *J* = 8.1 Hz, 2H), 3.91–3.83 (m, 2H), 3.62–3.54 (m, 2H), 1.32 (d, *J* = 6.8 Hz, 6H), 1.26 (d, *J* = 6.6 Hz, 6H), 0.83 (d, *J* = 6.3 Hz, 6H), −0.09 (d, *J* = 6.3 Hz, 6H). ^13^C NMR (126 MHz, CDCl_3_) *δ* [ppm] 169.11, 168.89, 166.72, 146.52, 143.78, 136.28, 132.67, 130.65, 128.12, 127.35, 126.32, 125.65, 125.64, 124.94, 121.0~121.8 (-CF_3_, q, *J* = 0.25 Hz), 119.98, 49.32, 47.56, 24.41, 24.32, 24.30, 22.88. MS (MALDI-TOF, *m*/*z*): [M^+^] calcd, 963.33; found, 963.77. Anal. Calcd for C45H46F6IrN5 (%): C, 56.12; H, 4.81; N, 7.27. Found: C, 56.14; H, 4.82; N, 7.25.

Synthesis of **(*p*-CF_3_piq)_2_Ir(dipba)** (abbreviated as ***p*-tFpqba**). This complex was collected as a dark-brown powder, according to the same procedure for ***o*-tFpqba** (193 mg, Yield: 41%). ^1^H NMR (500 MHz, CDCl_3_) *δ* 9.45 (d, *J* = 6.4 Hz, 2H), 8.92 (d, *J* = 8.2 Hz, 2H), 8.25 (d, *J* = 8.3 Hz, 2H), 8.08–8.04 (m, 2H), 7.83–7.77 (m, 4H), 7.76 (d, *J* = 6.5 Hz, 2H), 7.47 (t, *J* = 7.3 Hz, 2H), 7.41 (t, *J* = 7.3 Hz, 1H), 7.36–7.33 (m, 2H), 7.11 (d, *J* = 8.1 Hz, 2H), 6.55 (s, 2H), 3.29 (dt, *J* = 12.6, 6.3 Hz, 2H), 0.67 (d, *J* = 6.3 Hz, 6H), −0.14 (d, *J* = 6.2 Hz, 6H). ^13^C NMR (126 MHz, CDCl_3_) *δ* [ppm] 175.32, 169.02, 158.92, 149.74, 143.03, 136.66, 136.38, 130.77, 129.1~129.9 (-CF_3_, q, *J* = 0.24 Hz), 129.12, 128.44, 128.36, 128.31, 128.08, 127.82, 127.39, 126.57, 126.43, 120.81, 116.32, 48.27, 24.69, 24.48. MS (MALDI-TOF, *m*/*z*): [M^+^] calcd, 940.26; found, 940.66. Anal. Calcd for C45H37F6IrN4 (%): C, 57.50; H, 3.97; N, 5.96. Found: C, 57.47; H, 3.95; N, 5.98.

Synthesis of **(*p*-CF_3_piq)_2_Ir(dipg)** (abbreviated as ***p*-tFpqpg**). This complex was collected as a dark-brown powder, according to the same procedure for ***o*-tFpqpg** (230 mg, Yield: 48%). ^1^H NMR (500 MHz, CDCl_3_) *δ* [ppm] 9.26 (d, *J* = 6.4 Hz, 2H), 8.88 (d, *J* = 8.2 Hz, 2H), 8.23 (d, *J* = 8.3 Hz, 2H), 8.01 (d, *J* = 7.6 Hz, 2H), 7.79–7.72 (m, 4H), 7.63 (d, *J* = 6.4 Hz, 2H), 7.09 (d, *J* = 8.1 Hz, 2H), 6.49 (s, 2H), 3.90–3.82 (m, 2H), 3.61–3.53 (m, 2H), 1.31 (d, *J* = 6.8 Hz, 6H), 1.25 (d, *J* = 6.6 Hz, 6H), 0.84 (d, *J* = 6.3 Hz, 6H), −0.11 (d, *J* = 6.3 Hz, 6H). ^13^C NMR (126 MHz, CDCl_3_) *δ* [ppm] 169.08, 168.89, 160.31, 149.95, 143.81, 136.19, 130.57, 129.0~129.8 (-CF_3_, q, *J* = 0.24 Hz), 129.16, 128.38, 127.94, 127.30, 126.56, 126.40, 120.37, 116.11, 49.28, 47.48, 24.36, 24.26, 24.21, 22.87. MS (MALDI-TOF, *m*/*z*): [M^+^] calcd, 963.33; found, 963.73. Anal. Calcd for C45H46F6IrN5 (%): C, 56.12; H, 4.81; N, 7.27. Found: C, 56.11; H, 4.83; N, 7.26.

### 2.2. Photophysical Property

**Figure 1:**
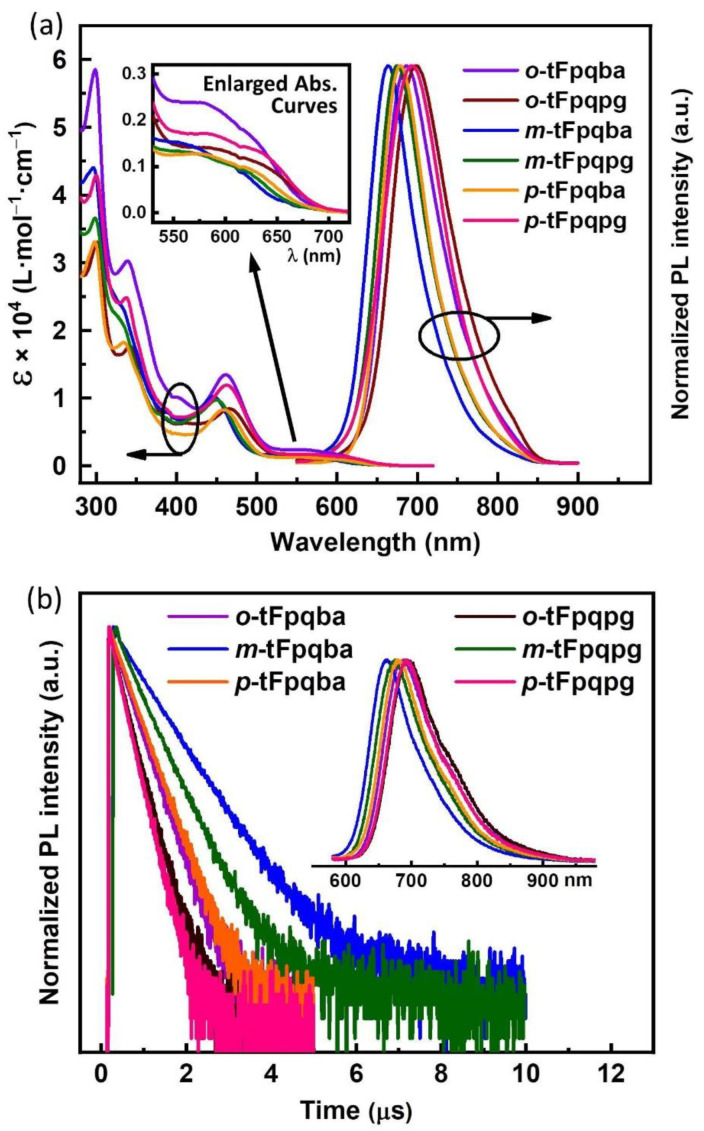
(**a**) UV–Vis absorption and emission spectra with 470 nm excitation in oxygen-free DCM (5 × 10^−5^ M) at 298K of all new complexes. Inset: enlarged absorption spectra in the range 550–700 nm. (**b**) Photoluminescence decay curves of the doped films based on Bebq_2_ doped with each complex (5 wt%). Inset: the corresponding PL spectra of these doped films with 365 nm excitation.

Figure 1a displays the UV–Vis absorption and photoluminescence (PL) spectra of six new phosphors in their CH_2_Cl_2_ dilute solution (5.0 × 10^−5^ M); a summary of the corresponding photophysical data is listed in Table 1. These spectral absorption features and assignments are similar to each other; all contain intense bands under 400 nm, which could be ascribed to their ligand-centered (^1^LC) π–π* transitions between the respective C^N ligands, and broad low-energy absorption bands that appear around 400–550 nm are likely to be ascribed to the spin-allowed metal-to-ligand charge-transfer (^1^MLCT) and ligand-to-ligand charge-transfer (^1^LLCT) transitions [7,8]. The weak absorption bands tailing beyond 550 nm are mainly assigned as admixtures of ^3^MLCT and ^3^LLCT as well as ^3^LC excited states, indicating the strong spin–orbit coupling of this new iridium complex family. From the normalized PL emission curves of these iridium (III) complexes, recorded in degassed CH_2_Cl_2_ (DCM) at room temperature, a common one-peak red emission in the 580–830 nm region with different maximum intensities at 684 nm, 697 nm, 663 nm, 676 nm, 679 nm, and 692 nm for *o*-tFpqba and *o*-tFpqpg is observed.

For *m*-tFpqba, *m*-tFpqpg, *p*-tFpqba, and *p*-tFpqpg, respectively, it is demonstrated that two meta-complexes have the shortest emission wavelength (663 and 676 nm) and two ortho-cases possess the longest *λ*_max_ values of 684 and 697 nm. On the other hand, three complexes adopting the dipg group as the LX ligand generally display a red shift of 13 nm relative to corresponding dipba-based counterparts with the same C^N ligands. This changing trend for emission color will be further verified in the following electrochemical test results. Such orderly and fine adjustment of the luminescence wavelength from 660 nm to 700 nm indicates that the molecular design strategy for tuning Ir (III) complexes’ emission color through synergistically considering the substituent effect of the C^N ligand and the electronic effect of the LX ligand is indeed practical and easily achievable. Furthermore, the vacuum-deposited films, by doping each complex in a well-matched energy gap host molecule bis(10-hydroxybenzo(h)quino-linato)beryllium complex (Bebq_2_) [15,19] with the same concentration of 5 wt%, show a single-peak emission from the respective dopant phosphors (Figure 1b), which exhibit a similar feather curve as the PL spectra of these phosphorescent molecules in the dilute solution and achieve fairly high quantum yield (PLQY) values between 0.15 and 0.37, indicating the efficient excited energy transfer from the Bebq_2_ to each dopant phosphor. Although the corresponding PLQYs of each recorded in solution are lower than 0.15 (Table 1) (which are reasonable according to the energy-gap theory) in solution, the low-energy triplet excited states of the deep red and/or NIR phosphors decay to their ground states through the non-radiative channels easier than those of the shorter-wavelength Ir complexes [9]. All phosphorescence lifetimes are generally shorter than 0.70 μs, suggesting that efficient spin–orbit coupling of these phosphors is beneficial for reducing efficiency roll-off in the corresponding PhOLEDs.

### 2.3. Electrochemical Properties

**Figure 2:**
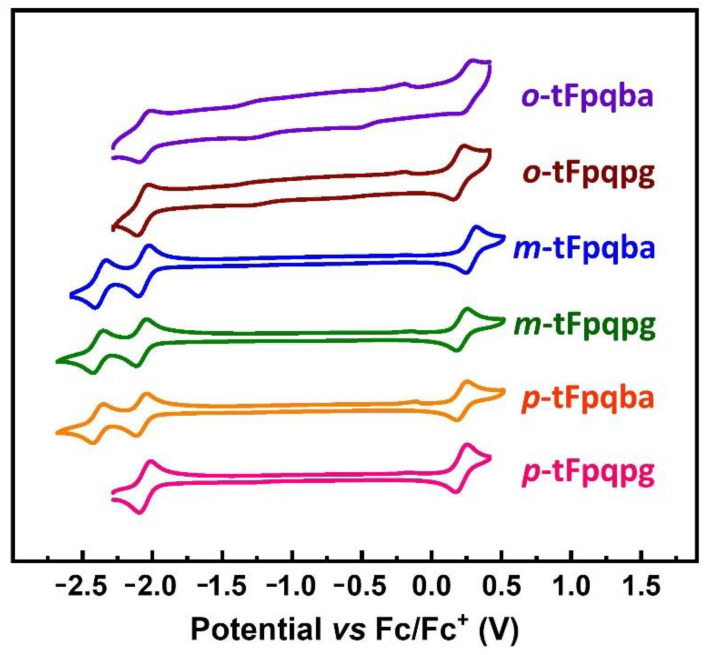
Cyclic voltammograms of all complexes recorded versus Fc^+^/Fc in newly distilled DMF solution with 0.1 M *n*-Bu_4_NPF_6_ supporting electrolyte and the scan rate being set to 100 mV s^−1^.

The electrochemical properties of these new complexes were explored by cyclic voltammetry (CV). Figure 2 shows the overlaid CV curves of the complexes recorded in DMF solution with ferrocenium/ferrocene (Fc^+^/Fc) coupling as the internal reference, which display both oxidation and reduction feature but are not, in some cases, completely reversible. As reported in other works in the literature, electrochemical testing is an experimental method to estimate the molecular HOMO and LUMO status. Although its accuracy is not as good as photoelectron spectroscopic methods (UV photoelectron spectroscopy (UPS) and inverse photoelectron spectroscopy (IPES)) [20], it is widely used in the field of optoelectronic materials because of its simple principle and convenience. As given in Table 2, all the LUMO energy levels estimated from the reduction potentials (vs Fc/Fc^+^) range from −2.80 to approximately −2.82 eV, indicating that the different positions of the –CF_3_ substituent on the ligands or ancillary ligands have no significant effect on their LUMO characters, whereas the HOMO energy levels of them are obviously affected by the varying chemical structure of the C^N together with the LX ligands. Compared to the dipba group, the stronger electron-donating ability of the dipg group makes three complexes based on dipg, being easily oxidized, leading to a cathodic shift in potentials as shown in Figure 2 [7]. As a result, the HOMO values are calculated to be −4.91, −4.94, and −4.93 eV for *o*-tFpqpg, *m*-tFpqpg, and *p*-tFpqpg, respectively, which are higher than their corresponding complexes by adopting the dipba group as the LX ligand in various degrees, resulting in narrower energy gaps of the -tFpqpg series than that of their –tFpqba counterparts. On the other hand, the oxidation potential of two phosphors with the electron-withdrawing group –CF_3_ meta-substituted in the phenyl moiety of C^N ligands, especially for *m*-tFpqba is clearly higher in comparison with the molecules containing the ortho- or para-position –CF_3_ group, which is effective for reducing the HOMO energy level and consequently widening the energy gap for two meta-substituted phosphors [21]. Therefore, the CV curves reflect the electrochemical behaviors of all these phosphorescent complexes, exhibiting a comprehensive effect originating from the different structures of C^N and LX ligands on the redox properties of complexes and, thus, leading to their different emission properties in terms of color emission, quantum yield, and excited-state lifetime.

### 2.4. Thermal Property

**Figure 3:**
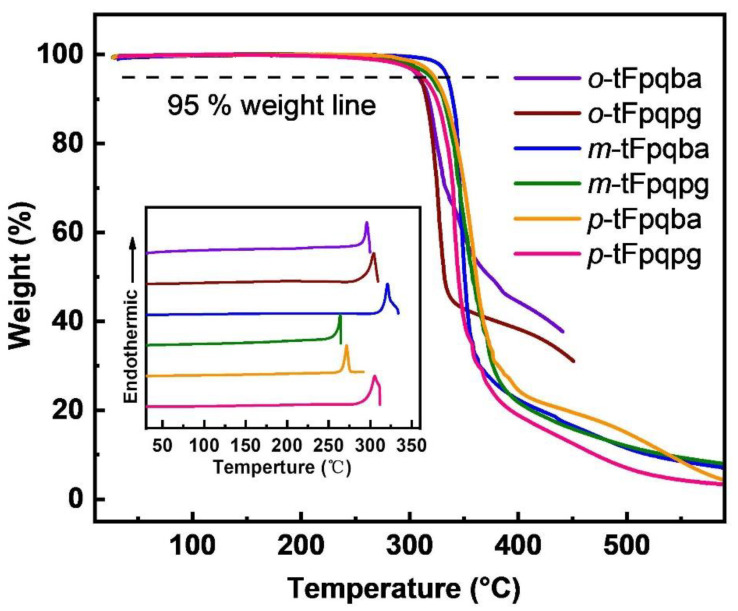
Thermal analyses for all complexes: TGA and DSC (inset) thermograms.

Six phosphorescent complexes were purified to >99.9% purity by vacuum sublimation, and the thermal properties were tested by thermos gravimetric analysis (TGA) and differential scanning calorimetry (DSC) under a nitrogen atmosphere (Figure 3 and Table 2). The decomposition temperatures of the complexes (*T*_d_, related to 5% weight loss) were over 300 °C and no obvious glass transition temperature was detected, which is beneficial for forming uniform evaporation film for the fabrication of OLEDs. This is quite an important and positive factor for improving the efficiency and lifetime of OLEDs.

### 2.5. Characterization of Phosphorescent OLEDs

**Figure 4:**
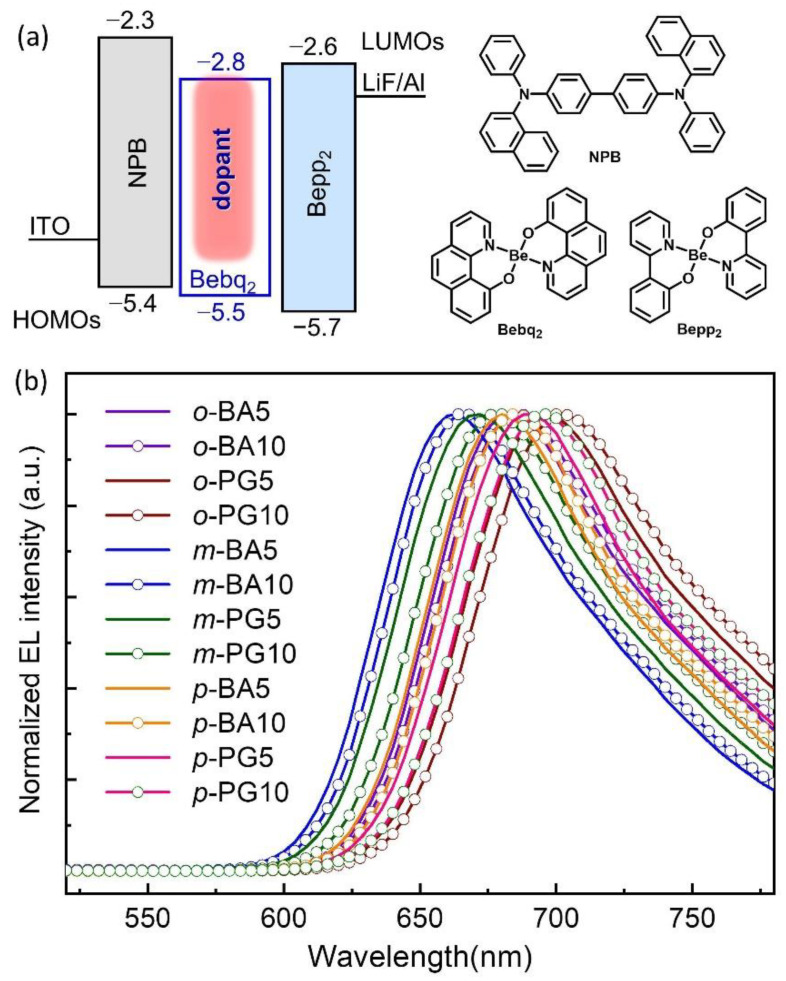
(**a**) Schematic energy level diagram of all the devices and chemical structures of various materials being used. (**b**) EL spectra of all the devices with the luminance of 100 cd m^−2^.

Based on these new phosphors, possessing similar (good) thermal stability, sufficient PL emission properties in solution, and doped thin films, a series of phosphorescent OLEDs, by employing doped films containing each dopant in a host molecule Bebq_2_ with two concentrations (5 and 10 wt%) as the emitting layers (EMLs) and adopting an optimized and simplified device configuration of [ITO/NPB (40 nm)/EML (20 nm)/Bepp_2_ (30 nm)/LiF (1 nm)/Al (150 nm), were fabricated (Figure 4a). Here, 4,4′-bis(N-(1-naphthyl)-N-phenylamino)biphenyl (NPB) served as a hole-transporting layer (HTL) and bis(2-(2-hydroxyphenyl)-pyridine)beryllium) (Bepp_2_) acted as an electron-transporting layer (ETL) [15,22]. Moreover, these PhOLEDs were named device *o*-BA5 and *o*-BA10, *o*-PG5 and *o*-PG10, *m*-BA5 and *m*-BA10, *m*-PG5 and *m*-PG10, *p*-BA5 and *p*-BA10, *p*-PG, and *p*-PG10, where the corresponding EMLs were six dopant emitters—*o*-tFpqba*, o*-tFpqpg, *m*-tFpqba, *m*-tFpqpg, *p*-tFpqba, and *p*-tFpqpg, respectively—doped in Bebq_2_ by adopting concentrations of 5 and 10 wt%. All devices achieved stable and intrinsic emission of each complex with a slight red-shift dependence on the concentration increase from 5% to 10% (Figure 4b). Moreover, they exhibited almost identical features to their PL spectra without any Bebq_2_ emission, indicating that all the EL emissions should be solely from the corresponding dopant phosphors through complete energy transfer between the host and dopant. As we expected, a combination of six phosphorescent-emitting dopants with two concentrations, being adopted here, led to 12 resulting PhOLEDs from fine tuning the luminescence color from 664 to 704 nm.

**Figure 5:**
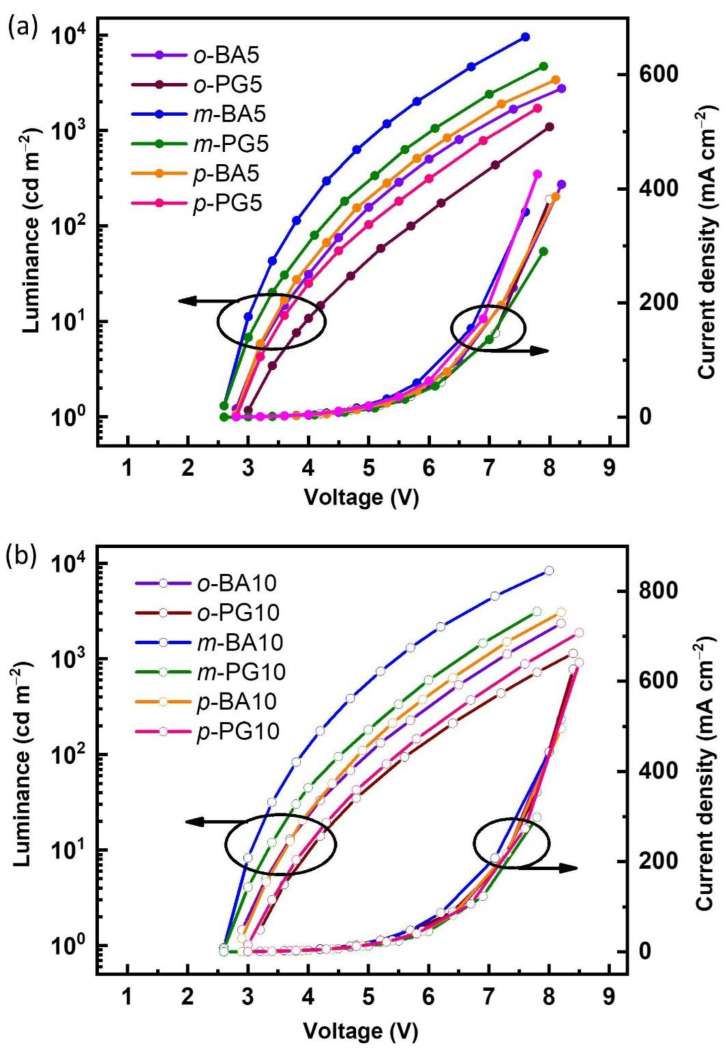
Current density–voltage–luminance (*J*–V–*L*) curves of series of devices with doping concentrations of 5 wt% (**a**) and 10 wt% (**b**).

**Figure 6:**
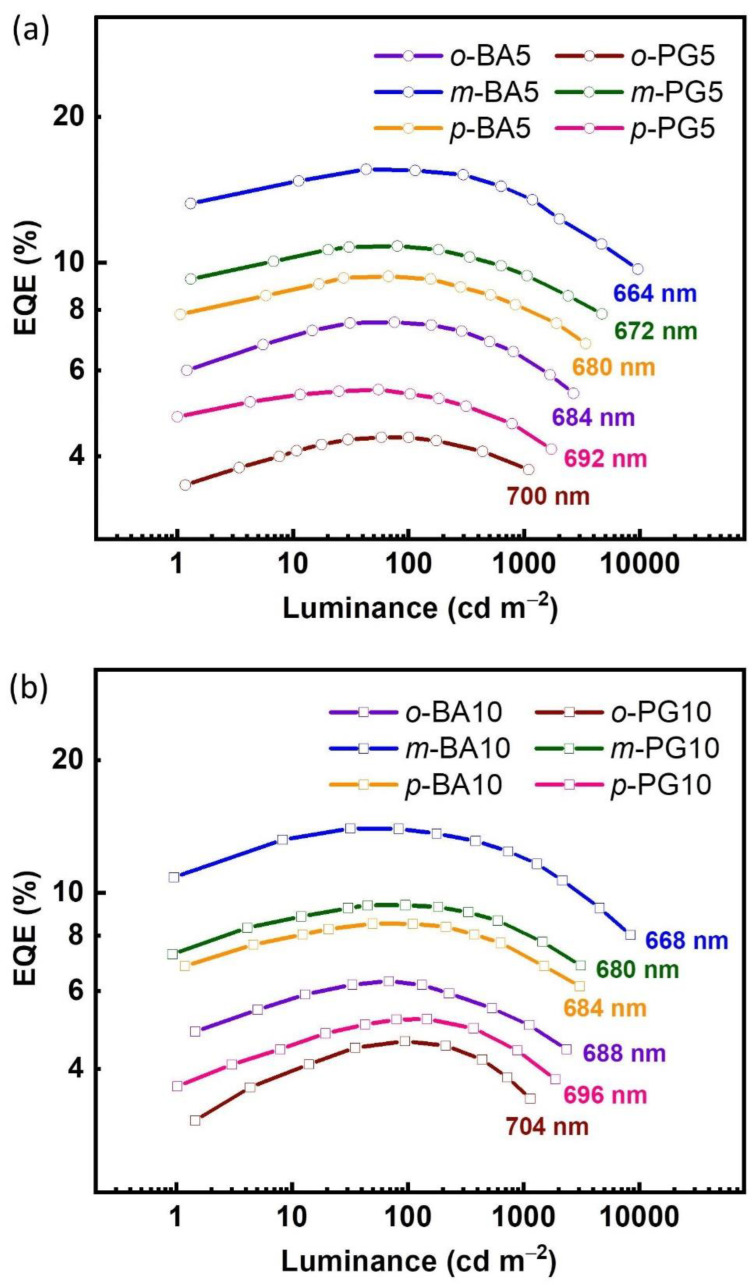
External quantum efficiency–luminance (EQE–*L*) curves of the devices with doping concentrations of 5 wt% (**a**) and 10 wt% (**b**).

The current density–voltage–luminance (*J*–V–*L*) and external quantum efficiency–luminance (EQE–*L*) characteristics of 12 devices are shown in Figure 5 and Figure 6, respectively, and the key EL data are summarized in Table 3. Generally, they displayed rather low turn-on voltages between 2.6 and 3.2 V, and their maximum luminance levels ranged from 1000 to nearly 10,000 cd m^−2^, showing an obvious dependence on the emission wavelength. That is, the shorter wavelength, lower driving voltage, higher luminance levels, and their EL efficiencies also show a similar trend. Among them, the four *m*-series devices based on the meta-substituted CF_3_ achieved higher EQEs, compared to the other two series of devices, where the highest performance was obtained by *m*-BA5 emitting a deep-red light with a peak emission of 664 nm. Moreover, it realized the highest luminance and EQE values based on the lowest driving voltage level, which maintained significantly high EQEs of 15.5% and 14.1% at a practical luminance of 100 and 1000 cd m^−2^ (or nit), respectively. Furthermore, as its counterpart device with 10% concentration, *m*-BA10 showed similar high efficiencies of 14.0% and 12.3% based on 100 and 1000 nit, respectively. This EQE level is much higher the theoretical maximum values estimated from the PLQY of *m*-tFpqba as shown in Table 1 [23], indicating that the host and dopant molecules should have a molecular orientation type conducive to light extraction in both the emitting systems by adopting concentrations of 5 and 10 wt% [24,25], which is beneficial to improve the out-coupling efficiency and the resultant high EL efficiencies. In fact, the study on molecular arrangement and orientation in thin film is in progress in our group, but due to the limitations of experimental conditions, no desirable result has been achieved yet. Along with the red-shift in the EL emission wavelength from *m*-series to *p*- and *o*-series devices, the EQE and luminance level continued to decline; however, two devices *o*-PG5 and *o*-PG10 with the longest wavelengths of 700 and 704 nm could still achieve EQEs as high as 4.3% and 4.6% at 100 nit, and 3.8% and 3.6% at 1000 nit, respectively.

According to the energy diagram, there is a slight energy offset between the HOMO levels of NPB and Bebp_2_, which implies that the process of hole injection from HTL (NPB) to the host molecules in EML needs to overcome a little injection barrier (~0.1 eV). Meanwhile, the HOMO-level difference between the dopants is about 0.5 eV, thus the phosphorescent molecules should play a role of hole charge trapping at a relatively low driving voltage. With the driving voltage increased, the higher voltages favor the injection of holes together with electrons into the EMLs through the host sites, which facilitates the creation of more excited states from Bebq_2_ molecules. The following EL processes were achieved through energy transfer from the host to each dopant; thus, the EQE levels (corresponding to the luminance) were mainly determined by the luminous efficiencies of the corresponding dopant films based on the Bebp_2_ host containing these phosphors, which are consistent with the above photophysical data in Table 1. On the other hand, the high overall EL performance level based on a phosphorescent complex family together with its near independence in the concentration range of 5–10% indicate that our molecular design strategy for developing efficient deep-red and NIR phosphors is indeed feasible and that the resulting phosphorescent complexes inherit certain bipolar properties originating from the high-quality four-membered Ir–N–C–N chelate ring constructed by the Ir (III) metal–ion center and the dipba and dipg LX ligands [13,14,15,16,17,18]. Correspondingly, these phosphorescent dopants can perform certain functions related to charge transport, acting as an additional auxiliary channel in conjunction with the Bebp_2_ host. This is in favor of balanced charge fluxes and broader recombination zones within the series of EMLs, effectively reducing triplet–triplet annihilation (TTA) processes, which are considered responsible for emitting state quenching, leading to unwanted EL efficiency roll-off [26]. These procedures ensure all these devices achieve, in general, high efficiency levels for deep-red and NIR emission OLEDs [2,8,19], especially under the practical luminance range of 100–1000 nit.

## 3. Materials and Methods

### 3.1. Photophysical Characterizations

UV–vis absorption spectra were recorded on the PE Lambda 1050 + UV/vis/NIR spectrometer with baseline already corrected. Steady-state emission spectra were recorded on the SHIMADZU 5301PC fluorescence spectrometer. Absolute photoluminescence quantum yields in doped films were recorded on an FLS920 spectrometer with an Xe light source through an integrating sphere (Edinburgh Instruments). The excited-state lifetimes were measured on an FLS980 spectrometer (Edinburgh Instruments) and analyzed using F980 software by reducing the chi-squared function (*χ*^2^). All samples were fresh and carefully prepared.

### 3.2. Electrochemical (Cyclic Voltammetry) and Thermal Properties

Electrochemical measurements were carried out on a BAS 100W Bioanalytical electrochemical workstation. A conventional three-electrode cell—Pt (work electrode), platinum wire (counter electrode), and porous glass wick Ag/AgNO_3_ (reference electrode) —was employed, and tetrabutylammonium hexafluorophosphate (*n*-Bu_4_NPF_6_, 0.1 M in DMF) was used as the supporting electrolyte. The scan rate was set to 100 mV s^−1^. The ferrocene/ferricenium (Fc/Fc^+^) couple was used as the internal standard. The HOMOs and LUMOs were calculated according to the formula: −[4.8 + E^ox/red^ − E(Fc/Fc^+^)] eV. Thermogravimetric analyses (TGA) were carried out on a TA Q500 thermogravimeter by measuring their weight loss while heating at a rate of 10 °C min^−1^ from 25 to 500 °C under nitrogen. Differential scanning calorimetric (DSC) measurements were performed on a DSC204 instrument (NETZSCH) at a heating rate of 10 °C min^−1^ from 20 to 460 °C under a nitrogen atmosphere.

### 3.3. Device Fabrication and Measurements

ITO glasses (sheet resistance of 20 Ω square^−1^) were pre-cleaned with a surfactant scrub followed by successive cleaning with deionized water, acetone, and isopropanol. After oxygen plasma cleaning for 5 min, ITO glasses were immediately transferred to the evaporation chamber and pumping vacuum. The organic was evaporingested under a pressure of around 2.6 × 10^−6^ Torr at a rate of 0.5 Å s^−1^, which is different from LiF (0.2 Å s^−1^) and Al (10 Å s^−1^). The active area, where the cathode overlaps with the anode, was 2 × 2.5 mm^2^. The thickness of the organic layers was monitored by a quartz crystal thickness/ratio monitor and was calibrated by the Dektak150 surface profiler (Veeco). The electrical characteristics and optical properties of devices were measured on a Keithley 2400 SourceMeter and PR655 spectrometer, respectively. The devices were not packaged, and the characterization processes were conducted under laboratory air conditions.

## 4. Conclusions

In summary, a series of novel Ir(C^N)_2_(L^X)-type heteroleptic iridium complexes emitting deep-red and NIR colors were synthesized, well characterized, and systematically investigated. Through introducing an electron-withdrawing group at different positions of the C^N ligands, combined crossly with varying electron-rich ancillary ligands, the opto-electronic characteristics of the target phosphorescent complexes in terms of emission color, frontier orbitals, and excited states were precisely and finely tuned by coordinately altering and matching the different C^N/L^X combinations. The PhOLEDs using resulting phosphors as dopant emitters achieved a significantly high overall EL performance level, especially under the practical luminance range of 100–1000 nit, which exhibited desirable independent properties in the concentration range of 5–10%. Thus, this work exhibits an easily accessible molecular design strategy for developing efficient deep-red and NIR phosphorescent emitters, which is indeed a feasible approach for enriching phosphorescent-emitting molecules for practical applications.

## Figures and Tables

**Table 1:** Photophysical data of all new complexes.

Complex	Abs. *λ*_max_ [nm] (ε × 10^4^ M^−^^1^ cm^−^^1^)	*λ*_PL_ [nm] Solution ^a^ /Film ^b^	*Φ*_PL_^a,b^ /τ_obs_^a,b^ [μs]
*o*-tFpqba	300(5.85), 342(3.03), 474(1.35)	684/688	0.05, 0.19/0.26, 0.37
*m*-tFpqba	297(4.40), 457(1.02)	663/663	0.12, 0.37/0.56, 0.69
*p*-tFpqba	300(3.31), 337(1.82), 471(0.83)	679/678	0.11, 0.26/0.33, 0.40
*o*-tFpqpg	302(3.30), 346(1.77), 478(0.85)	697/693	0.04, 0.15/0.13, 0.28
*m*-tFpqpg	300(3.66), 461(0.97)	676/675	0.10, 0.28/0.28, 0.50
*p*-tFpqpg	301(4.29), 342(2.48), 475(1.19)	692/693	0.06, 0.18/0.16, 0.25

^a^ Measured in oxygen-free dichloromethane solution at room temperature (5 × 10^−5^ M). ^b^ Measured in doped film based on Bebq_2_ host (5%) at room temperature.

**Table 2:** Electrochemical and thermal data of all new complexes.

Complex	Eox (V) ^a^	Ered (V) ^a^	HOMO/LUMO ^b^	*T*_d_^c^ [°C]
*o*-tFpqba	0.17	−1.98	−4.97/−2.82	310
*m*-tFpqba	0.21	−1.99	−5.01/−2.81	335
*p*-tFpqba	0.15	−2.00	−4.95/−2.80	323
*o*-tFpqpg	0.11	−1.99	−4.91/−2.81	310
*m*-tFpqpg	0.14	−1.99	−4.94/−2.81	319
*p*-tFpqpg	0.13	−1.98	−4.93/−2.82	312

^a^ Performed in DMF solvent with 0.1 M n-Bu_4_PF_6_ electrolyte with scan rate of 100 mV s^−1^ using a platinum wire as counter electrode and a porous glass wick Ag/AgNO_3_ as reference electrode. Potentials are referenced against the Fc^+^/Fc redox couple. ^b^ HOMOs and LUMOs are calculated according to the formula: −[4.8 + E^ox/red^-E(Fc/Fc^+^)] eV. ^c^ Corresponding to 5% weight loss.

**Table 3:** Summary of the EL performance for all devices.

Device	*V*_turn-on_ (V) ^a^	*L*_max_/cd m^−2^ (*V* at *L*_max_)	*EQE*^b^/%	*ELλ*_max_^c^/nm
*o*-BA5	2.8	2660 (8.2)	7.5, 6.4	684
*o*-BA10	2.9	2350 (8.2)	6.2, 5.0	688
*o*-PG5	3.0	1090 (8.0)	4.3, 3.8	700
*o*-PG10	3.2	1140 (8.4)	4.6, 3.6	704
*m*-BA5	2.6	9560 (7.6)	15.5, 14.1	664
*m*-BA10	2.6	8380 (8.0)	14.0, 12.3	668
*m*-PG5	2.6	4700 (8.0)	10.8, 9.3	672
*m*-PG10	2.6	3120 (7.8)	9.3, 7.7	680
*p*-BA5	2.8	3390 (8.1)	9.3, 8.1	680
*p*-BA10	2.9	3070 (8.2)	8.5, 7.0	684
*p*-PG5	2.8	1710 (7.8)	5.4, 4.6	692
*p*-PG10	3.0	1880 (8.5)	5.1, 4.2	696

^a^ Recorded at 1 cd m^−2^. ^b^ In order of the values at 100 and 1000 cd m^−2^. ^c^ Measured at 100 cd m^−2^.

## Data Availability

Not applicable.

## Sample Availability

Samples of all the compounds are available from the authors.
